# The molecular effect of metastasis suppressors on Src signaling and tumorigenesis: new therapeutic targets

**DOI:** 10.18632/oncotarget.5849

**Published:** 2015-09-27

**Authors:** Wensheng Liu, Zaklina Kovacevic, Zhihai Peng, Runsen Jin, Puxiongzhi Wang, Fei Yue, Minhua Zheng, Michael L-H. Huang, Patric J. Jansson, Vera Richardson, Danuta S. Kalinowski, Darius J.R. Lane, Angelica M. Merlot, Sumit Sahni, Des R. Richardson

**Affiliations:** ^1^ Molecular Pharmacology and Pathology Program, Department of Pathology and Bosch Institute, University of Sydney, Sydney, New South Wales, Australia; ^2^ Department of General Surgery, Shanghai Jiaotong University Affiliated First People's Hospital, Shanghai, Peoples Republic of China; ^3^ Department of General Surgery, Ruijin Hospital, Shanghai Jiao Tong University School of Medicine, Shanghai, Peoples Republic of China; ^4^ Department of Thoracic surgery, Ruijin Hospital, Shanghai Jiao Tong University School of Medicine, Shanghai, Peoples Republic of China

**Keywords:** metastasis suppressor, Src, NDRG1, metastasis

## Abstract

A major problem for cancer patients is the metastasis of cancer cells from the primary tumor. This involves: (1) migration through the basement membrane; (2) dissemination via the circulatory system; and (3) invasion into a secondary site. Metastasis suppressors, by definition, inhibit metastasis at any step of the metastatic cascade. Notably, Src is a non-receptor, cytoplasmic, tyrosine kinase, which becomes aberrantly activated in many cancer-types following stimulation of plasma membrane receptors (*e.g.*, receptor tyrosine kinases and integrins). There is evidence of a prominent role of Src in tumor progression-related events such as the epithelial–mesenchymal transition (EMT) and the development of metastasis. However, the precise molecular interactions of Src with metastasis suppressors remain unclear. Herein, we review known metastasis suppressors and summarize recent advances in understanding the mechanisms of how these proteins inhibit metastasis through modulation of Src. Particular emphasis is bestowed on the potent metastasis suppressor, N-myc downstream regulated gene 1 (NDRG1) and its interactions with the Src signaling cascade. Recent studies demonstrated a novel mechanism through which NDRG1 plays a significant role in regulating cancer cell migration by inhibiting Src activity. Moreover, we discuss the rationale for targeting metastasis suppressor genes as a sound therapeutic modality, and we review several examples from the literature where such strategies show promise. Collectively, this review summarizes the essential interactions of metastasis suppressors with Src and their effects on progression of cancer metastasis. Moreover, interesting unresolved issues regarding these proteins as well as their potential as therapeutic targets are also discussed.

## INTRODUCTION

Metastasis is a complex cascade process that involves a number of sequential events by cancer cells in order to “escape” from the primary tumor, penetrate tissue barriers, migrate to distant sites through the circulation and invade new organs (secondary site) to form new tumors [1]. However, the associated cellular, genetic and biochemical determinants in these processes are still largely unknown.

The viral *Src* (*v-Src*) gene encoded by the Rous sarcoma virus was the first defined oncogene and encodes the first recognized tyrosine kinase [2]. It can initiate and maintain cell transformation, even though it is irrelevant to viral replication [3]. Its cellular counterpart, c-Src, also plays a key role in tumorigenesis and metastatic progression [4, 5]. This latter molecule belongs to a family of non-receptor, membrane-associated, tyrosine kinases, including Fyn, Yes, Blk, Yrk, Fgr, Hck, Lck, and Lyn [4]. Importantly, c-Src is known to be over-expressed and/or hyper-activated in a wide variety of human cancers, which is caused by enhanced expression or dysregulation of upstream growth factor receptors and non-receptor tyrosine kinases, such as the epidermal growth factor receptor (EGFR), human epidermal growth factor receptor 2 (HER2), platelet-derived growth factor receptor (PDGFR), fibroblast growth factor receptor (FGFR), vascular endothelial growth factor receptor (VEGF), integrins, or focal adhesion kinase (FAK) [6–9]. Interestingly, over-expression of these receptors, their ligands, or both, is common in many tumor-types [10–12], and concurs with the fact that deregulation of c-Src tyrosine kinase activity occurs in various tumors, including those derived from the colon, pancreas, prostate, *etc*. [13–15]. Once activated, c-Src is involved in the regulation of oncogenic processes [16]. This, in turn, results in increased growth factor activity during tumorigenesis and the development of a metastatic phenotype [7].

Just as tumor promoters, such as oncogenic Ras or Src, play positive roles in regulating tumorigenesis, a growing body of literature demonstrates a new class of proteins, known as metastasis suppressors, that effectively inhibit metastasis [17, 18]. Examples of metastasis suppressors include Kangai1 (KAI1/CD82), E-cadherin, Rho GDP dissociation inhibitor 2 (RhoGDI2), Src-suppressed C kinase substrate (SSeCKS) and N-myc downstream regulated gene 1 (NDRG1) [19]. Interestingly, while these molecules are able to inhibit the formation of metastases, they generally do not affect formation of primary tumors [19, 20]. To achieve their anti-metastatic effects, these molecules regulate key cell signaling pathways, such as those involving Src, which directly influence cell motility and invasion [19]. The number of known metastasis suppressors continues to grow, but since most have only recently been discovered, their mechanisms of action are yet to be fully elucidated.

One metastasis suppressor that has recently attracted increasing interest, due to its potent anti-cancer effects, is NDRG1. This molecule was first identified as a tumor suppressor gene in human breast and prostate cancers [21], in which it was found to reduce cell growth both *in vitro* and *in vivo* [22–25]. Interestingly, over-expression of NDRG1 in breast, pancreatic and prostate cancer cell lines result in suppression of metastasis without suppression of tumorigenicity [26–28]. In clinical studies, NDRG1 was inversely correlated with breast and prostate cancer metastasis, while being positively correlated with patient survival [26, 28]. In our recent studies, as well as those from others, it has been demonstrated that NDRG1 plays a key role in the regulation of cellular signaling *via* a variety of pathways inhibiting cancer cell invasion and migration [23, 25, 29–31]. These signaling pathways include: (1) the phosphoinositide 3-kinase/protein kinase B (PI3K/AKT) and Ras/mitogen-activated protein kinase kinase (MAPK)/extracellular signal-regulated kinase (ERK) cascade [29, 32]; (2) the transforming growth factor-β (TGF-β) pathway [33], leading to the up-regulation of two key tumor suppressor proteins, namely phosphatase and tensin homolog deleted on chromosome 10 (PTEN) and mothers against decapentaplegic homolog-4 (SMAD4) [29]; (3) the Ras oncogenic pathway [29]; (4) β-catenin and the WNT pathway [25, 34]; as well as (5) the Rho-associated, coiled-coil containing protein kinase 1 (ROCK1)/phosphorylated myosin light chain2 (pMLC2) pathway [35].

In this review, we provide a perspective on NDRG1 and other metastasis suppressors, namely KAI1/CD82, E-cadherin, RhoGDI2 and SSeCKS, and the mechanisms involved in their interplay with the oncogene Src during tumorigenesis and metastasis. Moreover, we will discuss the rationale for targeting metastasis suppressor molecules such as NDRG1 as an emerging therapeutic modality.

## SRC KINASE

The most critical feature of tyrosine kinases is the strict regulation of their activity and functions [36]. Dysregulation of tyrosine kinase activity leads to progression of cancers [37]. Below, we briefly discuss the regulation of Src kinase activity and its role in tumor metastasis progression.

### The regulation of c-Src activity

**Figure 1:**
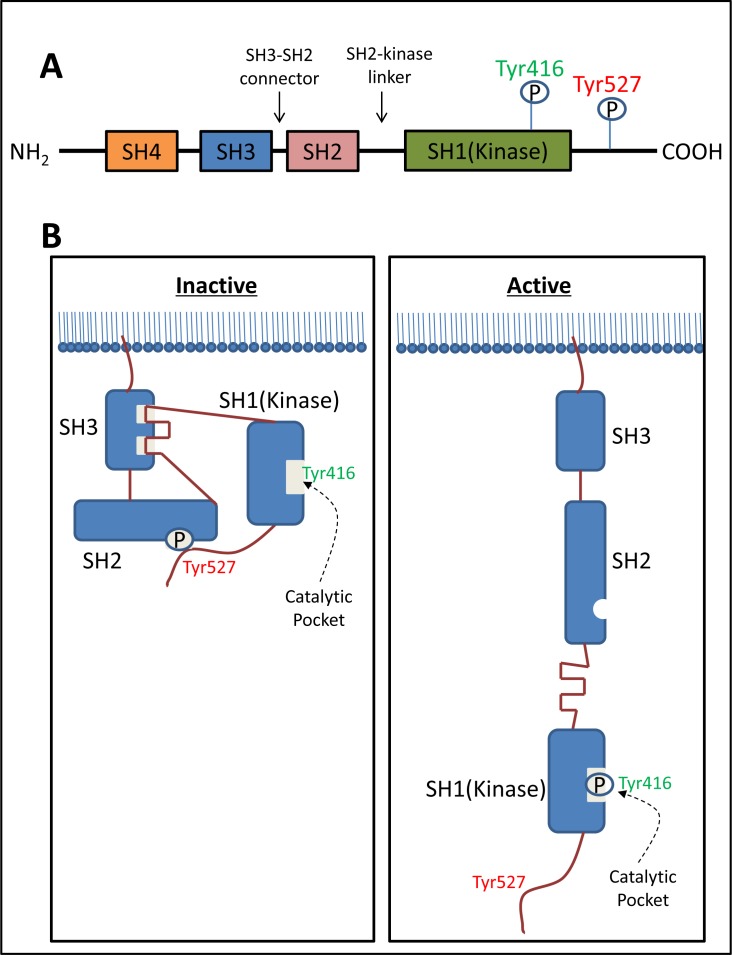
The structure of c-Src and regulation of its kinase activity **A.** Structurally, c-Src consists of a unique Src homology (SH) 4 domain, a SH3 domain, a SH3-SH2 connector, a SH2 domain, a SH2-kinase linker, a SH1-kinase domain, and a *C*-terminal tail regulatory region. **B.** The phosphorylation of two tyrosine sites (Tyr416 in the catalytic domain and Tyr527 in *C*-terminal region) and the intra-molecular interactions among the domains are crucial for the regulation of c-Src activity. Normally c-Src is present in its inactive form, in which Tyr527 is phosphorylated and stabilized by two intra-molecular interactions including: (1) binding of phosphorylated Tyr527 to its own SH2 domain; and (2) binding of the SH2-kinase linker to the SH3 domain. The de-phosphorylation of Tyr527 releases the ‘lock’ from the SH2 domain and causes dramatic conformational change in the kinase domain, subsequently catalyzing the intra-molecular auto-phosphorylation of Tyr416 in the activation loop.

Structurally, c-Src consists of a unique Src homology (SH) 4 domain, a SH3 domain, an SH3-SH2 connector, an SH2 domain, an SH2-kinase linker, an SH1 (kinase) domain, and a C-terminal tail regulatory region (Figure 1A) [36]. The phosphorylation of two tyrosine sites (Tyr416 in the kinase domain and Tyr527 in the C-terminal region) and the intra-molecular interactions among the domains are crucial for the regulation of c-Src activity (Figure 1B) [38].

c-Src is normally present in its inactive form, in which Tyr527 is phosphorylated and stabilized by two key intra-molecular interactions, including: (1) binding of phosphorylated Tyr527 to its own SH2 domain; and (2) binding of the SH2-kinase linker to the SH3 domain (Figure 1B) [39]. These intra-molecular interactions affect the configuration of the catalytic pocket (Figure 1B). The de-phosphorylation of Tyr527 releases the ‘lock’ by the SH2 domain and causes a dramatic conformational change in the kinase domain, subsequently catalyzing the intra-molecular auto-phosphorylation of Tyr416 in the activation loop (Figure 1B) [40]. This auto-phosphorylation locks the catalytic domain into the active conformation and facilitates access of substrates to the active site [40].

The regulation of c-Src activity by intra-molecular interactions suggests that it can also be regulated by interaction with molecules that compete with the functional domains. Indeed, c-Src binds to various tyrosine-phosphorylated proteins by recognizing specific phosphopeptide sequences via the SH2 domain [38]. For example, c-Src binds to the phosphorylated forms of p130Cas, FAK, and paxillin, as well as growth factor receptors such as EGFR, CSF-1R and PDGFR, resulting in activation of c-Src [5, 7, 41–43]. Activated c-Src can further phosphorylate these interacting proteins to create new binding sites for other adaptors and effectors, which in turn, allows amplification of down-stream signals [5, 44, 45]. In addition, the c-Src SH3 domain binds to various signaling proteins that contain proline-rich motifs, such as Shc, PI3K and p130Cas [46–48]. The interaction of c-Src with these proteins disrupts the stabilized inactive conformation of c-Src, resulting in the activation of this kinase and leads to its down-stream effects, which include the phosphorylation of these latter signaling proteins [36].

Although the molecular mechanisms of c-Src activation may vary depending upon the cell-type and extracellular stimuli, it is now believed that in general, full activation of c-Src is achieved by a series of events in the following order: (1) activated receptors such as PDGFR or EGFR, as well as protein tyrosine kinases such as FAK, recruit and interact with the SH2/3 domains of inactive c-Src to open the closed conformation; (2) tyrosine phosphatases de-phosphorylate the exposed pTyr527 to stabilize the active conformation; and (3) activated c-Src undergoes inter-molecular auto-phosphorylation on Tyr416 to lock the catalytic pocket into the fully active conformation [36, 38]. The fully activated c-Src can then phosphorylate substrate proteins, such as FAK and p130Cas, many of which can also create binding sites for c-Src which initiates the positive-feedback loop of c-Src activation [5]. When the cell response is terminated, activated c-Src is rapidly degraded *via* the ubiquitin-proteasome pathway [49], or inactivated by phosphorylation at Tyr527 [39] and dephosphorylation at Tyr416 [50, 51]. This highly secure regulatory system is required in order to strictly control potentially dangerous c-Src signaling, which possesses inherent oncogenic activity [36].

### The role of Src in cancer metastasis

During tumor progression, metastasis is an exceedingly complex process where primary tumor cells invade adjacent tissues, intravasate into the surrounding microvasculature and travel to distant sites where they may succeed in forming secondary tumors [52]. The increase of Src levels in metastasizing cancer cells compared to non-metastatic cells may represent an important step in the development of this more aggressive phase of cancer evolution [9, 53]. A series of studies have shown that Src activity increases with the progression of many cancers [15, 54–56]. In fact, Src activation has been used as a biological marker for tumor progression [54].

Src was found to play a key role in all steps of the metastatic cascade of colon cancer *via* its downstream targets [37, 57]. Specifically, Src promotes cancer cell detachment from the primary tumor by down-regulating the cell adhesion molecule, E-cadherin, and increasing matrix-degrading proteases (MMPs) [14, 58]. Moreover, it can also enhance cancer-cell focal adhesion *via* FAK and integrins (*e.g*., α5β1 integrin complexes; forming a focal adhesion complex) [41, 42]. Importantly, studies revealed that Src increases cell migration by modulating downstream effectors, such as p130Cas, PEAK, Cool-1, *etc*. [5, 53, 59–62]. Src is also involved in promoting angiogenesis, which is necessary to support the growth of secondary tumors [37]. This latter effect occurs through Src-mediated activation of STAT3 [63], which leads to increased expression of VEGF and interleukin-8 (IL-8), both of which are crucial for angiogenesis [52].

## THE INTERPLAY BETWEEN SRC AND METASTASIS SUPPRESSORS

Considering the growing advances in understanding the function of metastasis suppressors, as well as the vital role of Src in metastasis development, the interplay of these important molecules and how they affect cancer metastasis was important to examine. Hence, the metastasis suppressors, KAI1/CD82, E-cadherin, RhoGDI2, SSeCKS and NDRG1, and their interaction with Src, will be further discussed below.

### KAI1/CD82

KAI1/CD82 is a member of the tetraspanin family, which is known to be involved in the regulation of cell morphology, proliferation, fusion, motility and the immune system [64]. This protein plays an important role in cancer progression being initially identified as a metastasis-suppressor gene in prostate cancer [65]. Studies have shown that the expression of KAI1/CD82 is down-regulated in most metastatic cancers [64]. Its ability to inhibit cell motility, invasion and adhesion, together with the clinical observations that KAI1/CD82 expression is often lost/reduced in cancer, strongly suggests that KAI1/CD82 has an anti-oncogenic role in cancer [66]. Importantly, Src has been implicated to interact with KAI1/CD82 in the progression of several tumors and will be further discussed below.

### KAI1/CD82 suppresses the activity of Src

As indicated above, the activation of Src can be achieved by the interplay of growth factor receptors (EGFR, PDGFR, *etc*.) or integrins with Src [7, 43]. Integrins play a key role in cell-matrix adhesion and are responsible for mediating various signals from the cell surface to the extracellular matrix [67]. The promoter role of EGF during tumorigenesis has been very well recognized and its receptor (EGFR) is presently a target for many cancer therapies [10].

**Figure 2:**
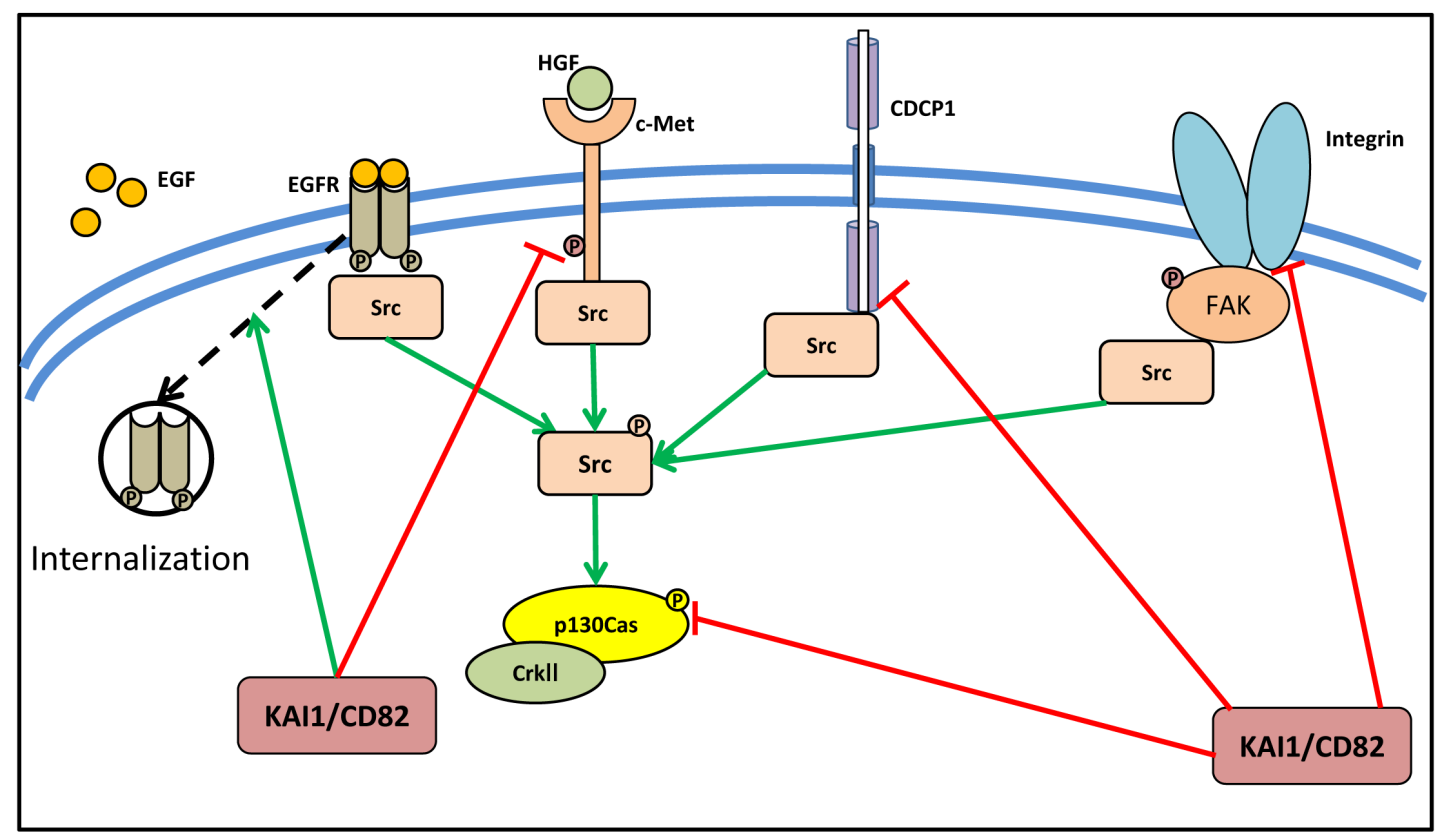
KAI1/CD82 suppresses the activity of Src *via* its upstream activator KAI1/CD82 plays an important role in cancer metastasis and was initially identified as a metastasis suppressor. KAI1/CD82 could attenuate EGFR signaling by promoting internalization of the activated receptor. It also reduces both integrin-dependent and HGF-induced activation of c-Met, subsequently inhibiting signaling to activate Src, resulting in reduced activation of p130Cas. Moreover, CDCP1, which promotes metastasis *via* enhancement of Src activity, was found to be suppressed by KAI1/CD82.

The association of KAI1/CD82 with integrins and EGFR has been extensively studied (Figure 2) [68, 69]. In fact, the activity of KAI1/CD82 in inhibiting cell motility and invasion is mediated through its ability to modulate the activity of receptor tyrosine kinases [64, 66, 69, 70]. It has been reported that KAI1/CD82 attenuates EGFR signaling by promoting internalization and subsequent degradation of the activated receptor (Figure 2) [69]. Another receptor tyrosine kinase that is suppressed by KAI1/CD82 is c-Met (also known as the hepatocyte growth factor receptor; HGFR), which is involved in oncogenic signaling in cancer cells (Figure 2) [68, 71]. In the prostate cancer cell lines, DU145 and PC3, KAI1/CD82 reduced both integrin-dependent and HGF-induced activation of c-Met, subsequently inhibiting signaling to activate Src, resulting in reduced activation of p130Cas [68]. Other than integrins and EGFR, KAI1/CD82 inhibits the expression of CUB-domain-containing protein 1 (CDCP1), which itself is known to be involved in the promotion of metastasis *via* enhancement of Src activity (Figure 2) [72]. Indeed, inhibition of CDCP1 expression *via* KAI1/CD82 in an *in vivo* tumor xenograft model, leads to significantly decreased levels of hypoxia-inducible factor-1α (HIF-1α) and one of its key down-stream targets, the angiogenesis promoting protein vascular endothelial growth factor-1 (VEGF-1) [72].

Moreover, KAI1/CD82 was found to have an important role in regulating cell-cell adhesion, again through its effects on inhibiting Src function [73]. Using the DU145 prostate cancer cell model, over-expression of KAI1/CD82 induced homotypic cell-cell aggregation [73]. This increase in cell-cell adhesion can be blocked by protein phosphatase 1, an inhibitor of Src kinase, or by the over-expression of a kinase negative Src mutant, indicating that the effect was mediated as a result of Src inhibition [73]. These novel findings indicate that KAI1/CD82 affects multiple targets to inhibit Src activity, which forms an integral part of its ability to function as a metastasis suppressor.

### KAI1/CD82 suppress the activity of downstream effectors of Src

Cell migration plays an essential role during cancer metastasis [74]. Recently, progress has been made in understanding the signaling pathways that control cell migration [75]. Signaling pathways directly down-stream of Src, which are mediated by p130Cas and CrkII, were found to determine directional persistence of cell migration by activating the small GTPases of the Rho family member Rac1, regulating actin re-organization, focal contacts, and membrane ruffling [76]. The small GTPases of the Rho family regulate multiple aspects of cell motility, such as generation of lamellipodia, assembly of focal adhesions, retraction of the cell tail, and formation of stress fibers by either directly acting on cytoskeleton reorganization or by cross-talk with the above signaling pathways [77, 78].

Importantly, both p130Cas and CrkII are involved in Src-mediated cancer cell invasion and migration [79, 80]. Using DU145 metastatic prostate cancer cells as the experimental model, it was demonstrated that inhibition of the p130Cas-CrkII pathway is crucial for the KAI1/CD82-mediated suppression of cell motility in DU145 cells [81]. In fact, KAI1/CD82 was found to reduce the level of p130Cas, and consequently, the coupling of phosphorylated p130Cas and CrkII required for cell motility [82], was also attenuated (Figure 2). The reduction in p130Cas was found to occur through post-transcriptional effects, as *p130Cas* mRNA level was not affected [81]. However, the precise mechanisms by which KAI1/CD82 were able to reduce p130Cas protein levels remains to be elucidated.

Collectively, KAI1/CD82 interacts with Src on multiple levels, playing an important role in inhibiting the activation of this latter oncogene, while also suppressing its downstream effects, ultimately leading to inhibition of cell motility, angiogenesis and metastasis.

### E-cadherin

Another metastasis suppressor that is involved in Src signaling is E-cadherin [83–85]. The human E-cadherin gene (located on 16q22.1) encodes a calcium-dependent, 120 kDa membrane protein that mediates cell-cell and cell-matrix adhesion [86]. E-cadherin is an epithelial adhesion molecule and plays a crucial role in maintaining the polarity of epithelial cells by preserving tight junctions and cytoskeletal systems [86, 87]. Increasing evidence indicates that E-cadherin is reduced in various tumor tissues, including colorectal cancer [88], breast cancer [89], prostate cancer [90], *etc*., compared with their corresponding normal epithelium. In addition, the loss of E-cadherin on the cell surface enables epithelial-derived cancer cells to transfer to a mesenchymal-like morphology, and thus, becoming more aggressive [91].

### Src inhibits the expression of E-cadherin

**Figure 3:**
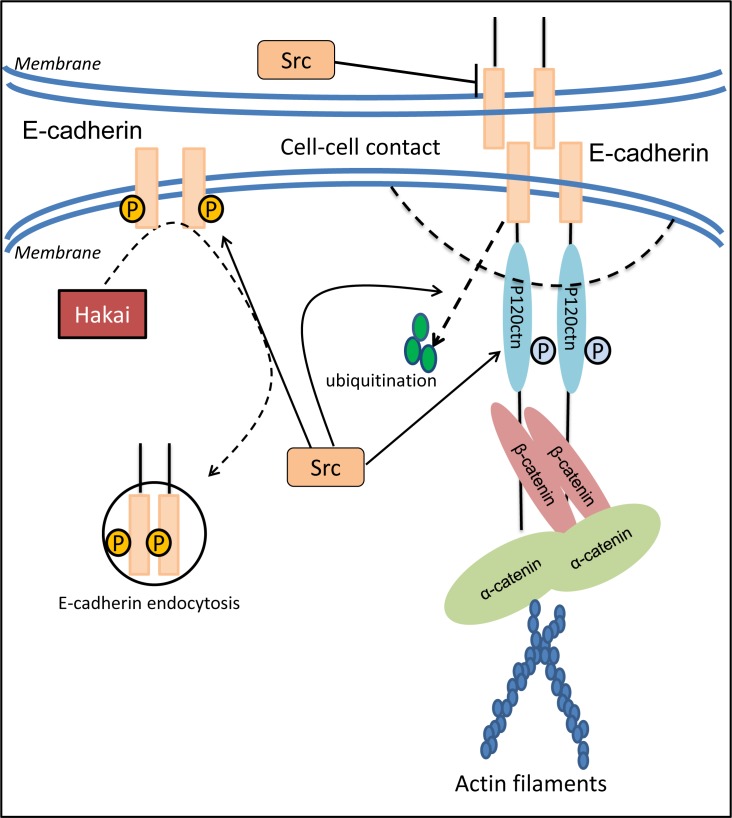
Src modulates cell adhesion through regulation of E-cadherin expression, distribution and function E-cadherin is one of the important epithelial adhesion molecules that plays a crucial role in maintaining the polarity of epithelial cells due to the disruption of tight conjunctions and reorganization of cytoskeleton systems. The decrease in E-cadherin based cell-cell contact induced by c-Src occurs not only through the prohibited expression of E-cadherin, but also *via* the enhanced endocytosis and further internalization of E-cadherin by regulating the E3 ligase, Hakai. Moreover, activated c-Src results in tyrosine phosphorylation of E-cadherin and p120-catenin. This effect leads to a weakened association between E-cadherin and p120-catenin that contributes to the instability of E-cadherin at the adherens junction, as well as E-cadherin ubiquitination and degradation.

Src and other Src-family kinases (SFKs) were found to inhibit the expression of E-cadherin (Figure 3), and thus, influencing cell-cell adhesion, cancer invasion and metastasis [92]. In fact, activated Src, *via* its SH2 and SH3 domains, induced the EMT by deregulating E-cadherin and inhibiting its function, while at the same time promoting assembly of integrin adhesion structures to promote a mesenchymal state [93]. Moreover, it was found that active Src caused components of the adherens junction to be re-distributed to Src-induced integrin adhesion complexes, leading to the conclusion that disruption of E-cadherin localization requires integrin signaling [84]. This was further confirmed by studies showing that E-cadherin redistribution was blocked by specific inhibitory antibodies to α or β integrin subunits [84]. In addition, another study indicated that the Src family inhibitor, protein phosphatase 2 (PP2), could enhance E-cadherin/catenin proteins and activate cell adhesion, which may lead to metastasis suppression [83].

Interestingly, E-cadherin can also be inhibited by other molecular pathways, namely TGF-β, although this latter effect was found to be independent of Src [94]. In fact, neither the specific Src family kinase inhibitor, SU6656, nor a dominant negative Src was able to inhibit TGF-β mediated EMT [94]. Hence, Src is not essential for the induction of the EMT, as this process is influenced by multiple signaling pathways [95, 96].

Paradoxically, while high levels of Src can inhibit the function of E-cadherin at the adherens junction, low levels of Src were found to play a positive supporting role on the function of this adhesion molecule [97]. Moreover, E-cadherin itself can also activate Src at cell-cell contacts, which then aids its own function [97]. This indicates that the interaction between E-cadherin and Src is complex and is mediated by both the levels of Src and their interaction at cell junctions.

The reduced levels of E-cadherin by Src are mediated not only through suppression of E-cadherin expression, but also *via* enhanced endocytosis and further internalization of this molecule (Figure 3) [87]. It was reported that when Src is activated in MDCK epithelial cells, the E-cadherin complex is ubiquitinated and endocytosed, and this was mediated by E-cadherin binding the E3 ligase, Hakai [98]. Once endocytosed, E-cadherin degradation was mediated by its shuttling from the endosome to the lysosome, a process that was mediated by hepatocyte growth factor-regulated tyrosine kinase substrate (Hrs) and Src-induced activation of the Rab5 and Rab7 GTPases [99]. The membrane redistribution of E-cadherin molecules engaged in mature junctions requires endocytosis and subsequent exocytosis [100]. Hence, the Src-mediated endocytosis of E-cadherin may directly decrease the distribution of E-cadherin on the epithelial membrane, and thus, stimulate tumor metastasis by disrupting cell-cell contacts.

Post-translational regulation of E-cadherin by Src-mediated phosphorylation is an essential requirement for endocytosis of E-cadherin [101]. The balance of degradation and re-expression after internalization are vital factors that affect protein levels and are responsible for rapid loss of E-cadherin expression [102]. Tyrosine phosphorylation of E-cadherin and its binding protein, β-catenin, was found to be strongly enhanced by Src [85]. Following the activation of tyrosine kinases, the tyrosine-phosphorylated E-cadherin complex attracts the E3 ligase protein, Hakai, resulting in its ubiquitin-dependent degradation and endocytosis (Figure 3) [98]. Tyrosine phosphorylation of E-cadherin was also found to reduce the association of this latter molecule with p120-catenin, contributing to the instability of E-cadherin at the adherens junction [103].

p120-catenin is a component of the cadherin adhesion complex being associated with E-cadherin, and is involved in the regulation of cadherin-mediated cell adhesion [104]. Interestingly, v-Src is able to phosphorylate p120-catenin in epithelial cells [105], which weakens its association with E-cadherin and subsequently affects E-cadherin-mediated cell adhesion (Figure 3) [106]. Considering that p120-catenin anchors E-cadherin to the actin cytoskeleton *via* α-catenin (Figure 3) [16, 106, 107], activation of SFKs disrupts these crucial bonds and leads to the rapid internalization of E-cadherin [16, 42, 95]. Hence, through its ability to inhibit E-cadherin expression and promote the degradation of this metastasis suppressor, Src is able to disrupt cancer cell adhesion and promote cell detachment, the initial step in the metastatic cascade.

### RhoGDI2

**Figure 4:**
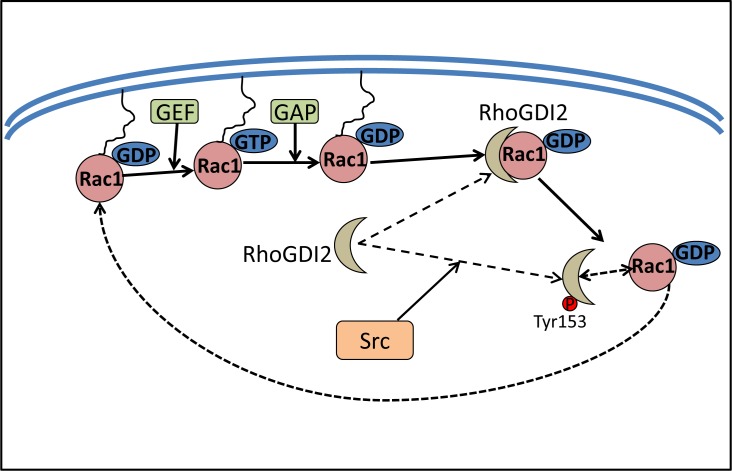
Src increases Rac1 activity by phosphorylating RhoGDI2 RhoGDI2 binds to Rho GTPases, sequestering them in the cytosol, which subsequently inhibits the activation of the Rho proteins and prevents their interaction with effectors or other regulatory proteins such as GAPs and GEFs. Src activation can modulate the RhoGDI2-Rho GTPase complex formation by phosphorylating RhoGDI2 at Tyr153, which could free Rac1 from its association with RhoGDI2. Subsequently, Rac1 then goes back into a functional cycle between GDP-bound Rac1 or GTP-bound Rac1.

RhoGDI2, also known as D4-GDI, Ly-GDI and ARHGDIB, has also been identified as a metastasis suppressor protein [108–110]. It belongs to a family of related proteins that also includes RhoGDI1 and RhoGDI3 [111]. RhoGDIs bind to Rho GTPases, namely Rac1, Rho and CDC42, sequester them in the cytosol, and maintain their GDP-bound inactive state [108]. This prevents their interactions with effectors or other regulatory proteins, namely GTPase activating proteins (GAPs) and guanine nucleotide exchange factors (GEFs; Figure 4) [108]. RhoGDI2 is ubiquitously expressed, and has been shown to interact with RhoA, Rac1, and Rac2 [108]. RhoGDI2 has been demonstrated to be a metastasis suppressor in different cancers, as its expression was decreased or lost in metastatic cancers, including bladder cancer and Hodgkin's lymphoma [109, 112]. Paradoxically, RhoGDI2 has also been shown to promote metastasis in other cancers, namely ovarian adenocarcinoma, breast cancer, *etc*. [113, 114]. These functional differences may be due to cell type specificity, variations in the experimental approaches, patient populations, and statistical analyses.

### Src and RhoGDI2

The phosphorylation of RhoGDI1 and RhoGDI2 by Src was shown to inhibit the ability of these former proteins to complex with Rho GTPases, allowing these latter proteins to become active [115]. In fact, RhoGDI2 phosphorylation by Src decreased its association with Rac1, leading to an increase of the active Rac1-GTP (Figure 4). Interestingly, phosphorylation of RhoGDI2 by Src at Tyr153 and, to a lesser degree, Tyr24, not only decreased the amount of Rac1 in RhoGDI2 complexes, it also increased RhoGDI2 association with cell membranes [116]. The function of RhoGDI2 at the cell membrane remains to be elucidated, although it has been speculated to contribute to its anti-metastatic effects [116].

Rho GTPases play a critical role in cellular activities, including growth and differentiation, apoptosis, cell motility, and various other aspects of cytoskeletal dynamics and cell polarity [117, 118]. Hence, through its ability to phosphorylate RhoGDI2, Src is able to promote the activation of Rho GTPase proteins and subsequently promotes metastatic progression in cancer cells.

### SSeCKS

Src-suppressed C kinase substrate (SSeCKS) is a metastasis suppressor that is also known as the ortholog of human GRAVIN/AKAP12 [119]. It was originally identified in a screen for genes markedly down-regulated by v-Src [120]. Importantly, there is increasing evidence suggesting that SSeCKS is reduced in metastases when compared to the primary tumors in a number of neoplasms, including prostate, breast, *etc*. [121–123]. Moreover, SSeCKS has been utilized as a predictive marker for prostate cancer metastasis [122]. These studies indicate that loss of SSeCKS expression is correlated with increased metastatic potential of human malignancies.

Current evidence suggests that SSeCKS functions as a scaffold protein, which controls mitogenic signaling and cytoskeletal remodeling by binding key signaling mediators such as PKC, PKA, calmodulin, F-actin, cyclins, Src and phospholipids in a spatiotemporal manner [124]. SSeCKS also participates in the control of cytoskeletal reorganization associated with motility, which is most likely facilitated by domains that link it to both plasma membrane and cytoskeletal sites [125].

### Src suppresses the expression of SSeCKS

SSeCKS was found to be down-regulated by several oncogenes (*Ras, Src, Myc, Jun, Fos, Wnt1, etc*.) in various cancers including prostate, lung, gastric, breast and ovarian [121–123, 126–128]. However, the mechanism by which SSeCKS is down-regulated by Src still remains unclear. The fact that SSeCKS is down-regulated by specific group of oncogenes like *Src, Myc, Jun, etc*., but not other oncogenes such as *Raf, Mos*, or *Neu*, suggests that it is controlled by specific mitogenic and oncogenic pathways [129].

Studies have elucidated several mechanisms by which SSeCKS is transcriptionally regulated. In humans, SSeCKS transcription is driven by three independent promoters, which encode for three different *SSeCKS* transcripts, namely α, β and γ [130]. v-Src was found to repress *SSeCKS* transcription through its effects on the E- and GC-boxes in the *SSeCKS α* proximal promoter, which are bound by the transcription factors, USF1 and SP1/3, respectively [131]. In fact, v-Src promoted the complex formation between USF1 and SP1/3, increasing the binding of SP1/3 to the *SSeCKS α* promoter [131]. This led to the recruitment of HDAC, which prompted a chromatin structure change that affected both the α and the down-stream β promoters and resulted in suppressed SSeCKS expression [131]. Another study revealed that the transcription factor, TFII-I, which converts to a transcriptional repressor once it has been at phosphorylated at Tyr248 by activated Src, also plays an important role in the Src-induced suppression of *SSeCKS* transcription [132].

### SSeCKS inhibits Src oncogenic effects *via* disengaging active Src from its down-stream effectors

Interestingly, while Src can inhibit SSeCKS transcription, this latter metastasis suppressor can also inhibit Src activity by disrupting the link between Src and its down-stream mediators [133, 134]. In fact, SSeCKS inhibits oncogenic motility and invasiveness by disengaging growth factor-activated Src from activating the PKC-Raf-MEK-ERK pathways that control the formation of podosome/invadosome structures and promote the expression/secretion of MMPs [124, 135]. This, together with the fact that SSeCKS alters the actin-based cytoskeletal architecture [125], suggests that SSeCKS inhibits Src oncogenic signaling by physically sequestering it away from downstream signaling mediators.

**Figure 5:**
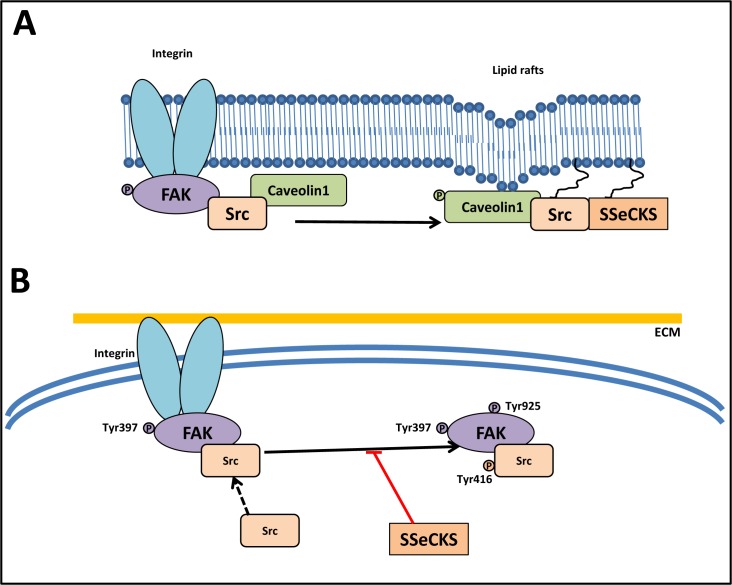
Src abrogates the function of the FAK/Src complex **A.** SSeCKS attenuates the ability of Src to induce metastatic progression by directly scaffolding Src away from FAK complexes to lipid rafts (caveolae structures). SSeCKS facilitates Src association with caveolae structures by mediating Tyr14 phosphorylation of caveolin-1. **B.** SSeCKS effectively suppress the formation of constitutive FAK/Src complexes and FAK activation (Tyr397 and Tyr925 phosphorylation) that promote oncogenic down-stream signaling pathways.

The ability of SSeCKS to sequester Src from FAK, which play a crucial role in mediating signaling to the actin-based cytoskeleton, inhibits the FAK/Src complex (Figure 5A) [133]. In fact, SSeCKS directly sequesters Src pools from FAK complexes to lipid rafts in the plasma membrane, attenuating the ability of Src to induce metastatic progression (Figure 5A) [133]. SSeCKS also suppressed adhesion-induced Src activation (phosphorylated Src at Tyr416) and phosphorylation of FAK at Tyr925, a known Src substrate site [133].

Another mechanism by which SSeCKS might disengage active Src from down-stream oncogenic signaling is based on the identification of a Src scaffolding domain in SSeCKS, which is homologous to the Src-binding domain in Caveolin-1 (Figure 5B) [133]. A recent model suggested that SSeCKS enhanced relative adhesion-induced FAK phosphorylation levels at Tyr397, yet suppressed phosphorylation at Tyr925, suggesting that Src is disengaged by SSeCKS from normal FAK/Src complexes. Direct binding between Src and SSeCKS *via* a domain homologous to the Src-binding site on Caveolin-1 was observed [133]. Hence, SSeCKS attenuates Src's ability to induce metastatic progression by directly sequestering Src from its down-stream targets.

Using cDNA microarrays and semi-quantitative RT-PCR analysis, it was found that SSeCKS re-expression resulted in the attenuation of critical Src-induced proliferative and pro-angiogenic genes including *Afp, Hif-1α, Cdc20a* and *Pdgfr-β* [134]. Conversely, SSeCKS induced several cell cycle regulatory genes such as *Ptpn11, Gadd45a, Ptplad1, Cdkn2d* (p19), and *Rbbp7* [134].

Together, these studies indicate that SSeCKS can suppress Src-induced oncogenesis by modulating gene expression down-stream of Src kinase activity.

### NDRG1

NDRG1 (also known as Drg1, RTP, Rit42, PROXY-1 or Cap43) belongs to the human NDRG family, which also comprises NDRG2, NDRG3 and NDRG4 that share a 53–64% amino acid identity with each other [21, 136–138]. The *NDRG1* gene has been mapped to chromosome 8q24.3 (8q24.2 in the AceView database [139]) [140] and encodes a stable protein which is ubiquitously expressed and predominantly cytosolic [24].

### Functions of NDRG1 in cancer

NDRG1 is a multifunctional protein involved in tumorigenesis and tumor development, and its function differs in different tumor-types [22, 24, 141]. In colorectal, prostate, cervical, and ovarian cancers, NDRG1 plays important roles in preventing tumor progression and metastasis, which suggests that NDRG1 has a role as a tumor suppressor, metastasis suppressor, or both in these cancers [23, 142–144]. However, in hepatocellular carcinoma, NDRG1 enhances portal vein invasion and intra-hepatic metastasis, indicating that this protein plays pleiotropic roles, with its activity being context-dependent [145]. Hence, it is clear that NDRG1 plays an important role in the promotion or inhibition of carcinogenesis depending on factors such as the cell-type.

NDRG1 has been demonstrated to inhibit primary tumor growth *in vitro* and *in vivo* [21, 146]. *In vitro* studies demonstrated that over-expression of NDRG1 significantly decreased the proliferation rate of MCF7 breast and EJ bladder cancer cell lines [21]. Moreover, these cells were also found to form smaller colonies on soft agar relative to control cells [21]. Further, mice injected with NDRG1 over-expressing EJ bladder cancer cells exhibited smaller tumors compared to those injected with control EJ bladder cancer cells [21]. Subsequent studies reported a reduction in tumor microvascular density, invasion depth and histopathological grading, with a corresponding increase in overall survival rates for pancreatic cancer patients with higher levels of NDRG1 expression [27]. Interestingly, in this latter study, although NDRG1 over-expression reduced tumor growth *in vivo*, cell growth was not affected *in vitro*, potentially due to *in vivo* modulatory factors such as those associated with the stroma and angiogenesis [27].

The establishment of metastatic lesions is dependent upon successful initiation of angiogenesis, a process that is essential for providing the oxygen and nutrients required for cell growth. Interestingly, NDRG1 was found to inhibit the process of angiogenesis by negatively regulating critical pro-angiogenic factors, such as IL-8, MMP-9 and VEGF1, in pancreatic cancer [27]. Moreover, NDRG1 has been shown to suppress angiogenesis *via* attenuating the expression and phosphorylation of the inhibitor of κB kinase (IκBα) and subsequently NF-κB signaling [147].

In addition to its effects on primary tumor growth, the role of NDRG1 as a metastasis suppressor has been demonstrated *in vitro* [26, 28] and *in vivo* [25, 31, 146, 148]. NDRG1 was shown to inhibit metastasis by decreasing cell–cell and cell–matrix adhesion in AT6.1 rat prostate cancer cells [149] and to inhibit metastasis to lungs without affecting primary tumor growth in a SCID mouse model [26]. NDRG1 expression was also found to inhibit cell proliferation in the metastatic colonic cancer cell line, HCT116 [150]. Further, suppression of NDRG1 was demonstrated to significantly enhance cell proliferation, migration and invasion in Ishikawa endometrial cancer cells [151]. In contrast, over-expression of NDRG1 was shown to inhibit cellular proliferation and migration of this latter cell line [151].

Recently, NDRG1 has been shown to suppress metastasis by a mechanism involving the modulation of the structural protein actin [35]. In cancer cells, actin is polymerized to form stress fibers that are required for cell migration [152]. NDRG1 has been demonstrated to inhibit the Rho-associated, coiled-coil containing protein kinase 1 (ROCK1)/phosphorylated myosin light chain 2 (pMLC2) pathway [35], which would result in suppression of the assembly and rearrangement of stress fibers from actin [153]. Furthermore, NDRG1 modulates metastasis *via* proteins including MMPs, which degrade extracellular matrix, and adhesion molecules, such as β-catenin and E-cadherin that form the adherens junction at the cell membrane [33, 34, 154]. In agreement with these latter studies, NDRG1 has also been demonstrated to promote the membrane expression of β-catenin in breast, prostate and colon cancer cells [25, 34]. Further, NDRG1 has also been shown to inhibit the TGF-β-induced EMT and to restore membrane β-catenin and E-cadherin levels, which are suppressed by TGF-β in cancer cells [33]. Together, these observations indicate that NDRG1 promotes the formation of the adherens junction, which is critical for cell–cell adhesion, and elucidates the mechanisms by which this molecule is able to suppress metastasis in cancer cells.

### Src and NDRG1 interactions

**Figure 6:**
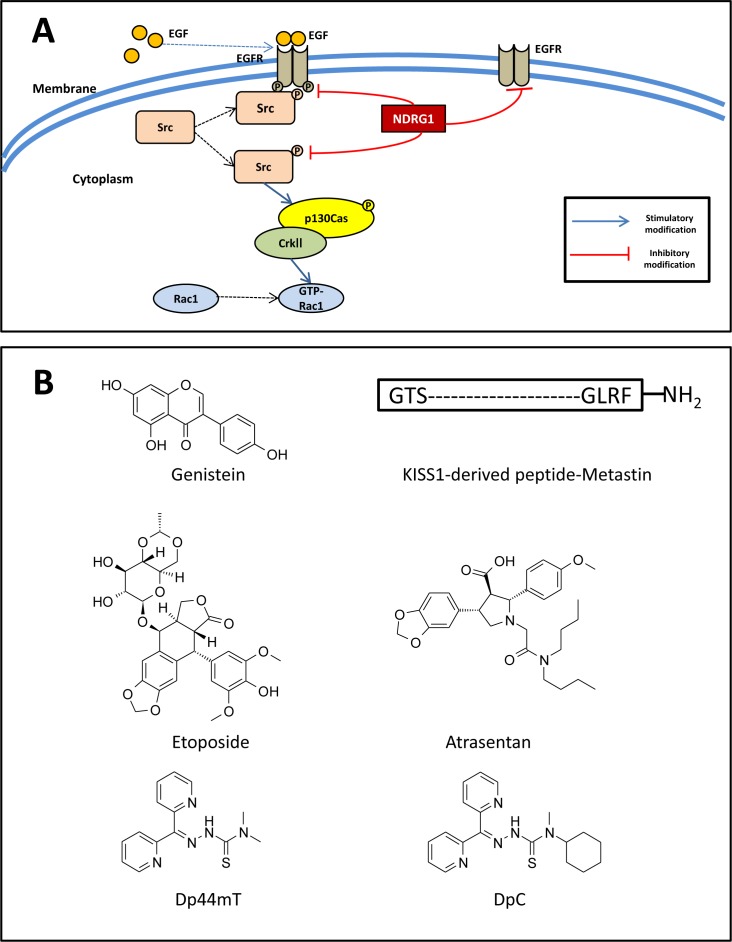
**A.** NDRG1 inhibits Src activity and its downstream signaling pathway. **B.** Line drawings of the structures of potential anti-metastatic agents under development. **A.** NDRG1 expression inhibits c-Src phosphorylation at its activating site (Tyr416). This occurs through NDRG1-induced reduction in EGFR expression, abrogation of EGF-mediated EGFR activation, and thus preventing the EGFR-c-Src interaction. Moreover, NDRG1 was shown to suppress Rac1 activity by modulating the phosphorylation of a c-Src down-stream effector, namely p130Cas and its association with CrkII, which acts as a molecular switch to activate Rac1. **B.** Line drawings of: Genistein, KISS1-derived peptide, etoposide, atrasentan, di-2-pyridylketone 4,4,-dimethyl-3-thiosemicarbazone (Dp44mT) and di-2-pyridylketone 4-cyclohexyl-4-methyl-3-thiosemicarbazone (DpC).

Considering the significant roles of both NDRG1 and Src in cancer metastasis [9, 24, 29, 34, 155], we recently conducted studies that explored the potential interplay of these two molecules [156]. It was discovered that in both prostate cancer DU145 and colon cancer HT29 cell models, NDRG1 over-expression significantly decreased Src phosphorylation at Tyr416, while it had no significant effect on Src phosphorylation at Tyr527 or total c-Src levels (Figure 6A) [156]. Further, incubation of these cells with the EGF ligand revealed that NDRG1 affected c-Src activation *via* decreasing EGFR expression, leading to loss of activated EGFR, and thus, preventing the EGFR-c-Src interaction (Figure 6A) [156].

Moreover, a key substrate and down-stream effector of Src, namely p130Cas, was also inhibited by NDRG1 expression [156]. That is, over-expression of NDRG1 markedly reduced the phosphorylation of p130Cas at Tyr249 and Tyr410 (Figure 6A), both of which are located in the substrate-binding domain and are vital for p130Cas activation [80, 157]. Importantly, the modulation of p130Cas activation by NDRG1 occurred in a Src-dependent manner, as transient silencing Src expression or pharmacologically inhibiting Src activity reversed the inhibitory effect of NDRG1 on p130Cas [156]. As described above, phosphorylation of p130Cas promotes its binding to CrkII, which subsequently recruits DOCK180, leading to the activation of the Rho family GTPase Rac1 [156]. In fact, as a result of its effect on p130Cas, NDRG1 was also able to suppress Rac1 activity, as demonstrated by a Rac1 activation assay assessing the levels of GTP-bound Rac1 (GTP-Rac1; Figure 6A) [156]. When migration assay experiments were performed, it was shown that NDRG1 reduced cancer cell migration through inhibition of Src activation [156].

Considering its marked anti-metastatic activity, NDRG1 presents a promising molecular target for anti-metastatic agents. In fact, a novel class of pharmacological agents were found to significantly up-regulate NDRG1 expression in a range of neoplasms [23, 32, 158, 159]. As a result, these agents were also found to decrease Src activation [156]. These recent findings highlight the potential of metastasis suppressors as novel therapeutic targets and this is further discussed below.

## METASTASIS SUPPRESSORS AS THERAPEUTIC TARGETS

Identification of proteins that inhibit dissemination of cancer cells will provide new perspectives to define novel therapeutics. Based on the function of metastasis suppressor genes in cancer regression, they have become a hot topic for therapeutic approaches. Several strategies have been developed to potentiate the expression of metastasis suppressors. These strategies include direct administration of the gene product, re-expression of the endogenous locus, restoration of function by gene therapy, and identification of downstream effectors associated with the loss of metastasis suppressor proteins, which have been summarized [19].

Development of anti-metastatic drugs that trigger or mimic the effect of metastasis suppressors represents new therapeutic approaches to improve patient survival [19]. A number of drugs that can restore or mimic the effect of target proteins have proven promising in preclinical and clinical studies. For example, genistein (Figure 6B), an agent for re-induction of KAI1/CD82, was shown to inhibit the invasive behavior of prostate tumor cells in nude mice [160]. Moreover, administration of the KISS1-derived peptide, Metastin (Figure 6B), demonstrated promising anti-metastatic effects on melanoma cells in a preclinical study [161].

There are also other examples of re-expression or induction to develop therapies targeting metastasis suppressor genes. As mentioned above, KAI1/CD82 acts as metastasis suppressor in many malignant tumors [64, 66]. Several strategies have been employed to re-express KAI1/CD82 in cancer cells *via* targeting its transcription [162, 163]. The tumor suppressor, p53, has been demonstrated to increase transcription of KAI1/CD82 through a p53-responsive element [162]. This concept led to the use of etoposide (Figure 6B), an agent that induces p53, and increases KAI1/CD82 expression in prostate cancer [163]. Another potential therapeutic agent, namely atrasentan (Figure 6B), which is currently in Phase III trials for stage IV prostate cancer, was shown to antagonize endothelin 1, a down-stream molecules of metastasis of RhoGDI2, mimicking the role of RhoGDI2 and reducing T24T cell metastases in animal models [164, 165].

A novel class of anti-cancer agents currently under development are the thiosemicarbazones [166–168], that selectively target cancer cells based on their increased requirements for iron [169–172]. Iron plays a crucial role in proliferation and DNA synthesis and neoplastic cells have an increased requirement for iron as shown by their markedly elevated expression of the transferrin receptor 1 and enhanced uptake of iron [171, 173, 174]. Novel thiosemicarbazones bind iron and copper in cancer cells and also form redox active complexes which results in multiple down-stream effects [175–177] and alter the expression of a variety of proteins involved in cell cycle control, such as members of the cyclin family and cyclin-dependent kinases [166, 178, 179]. Importantly, these agents also up-regulate the growth and metastasis suppressor protein, NDRG1 [158], which has been shown to be vital in the progression and outcome of a variety of neoplasms [24, 25, 31, 180–182], as described above. Iron chelators up-regulate NDRG1 *via* HIF-1α-dependent and -independent mechanisms [158], with iron depletion being required for this effect to occur [158, 178].

A variety of chelators have been developed, with ligands of the di-2-pyridylketone thiosemicarbazone (DpT) class, including di-2-pyridylketone 4,4-dimethyl-3-thiosemicarbazone (Dp44mT; Figure 6B) and di-2-pyridylketone 4-cyclohexyl-4-methyl-3-thiosemicarbazone (DpC; Figure 6B), demonstrating the most potent and selective anti-cancer activity both *in vitro* and *in vivo* against a range of different tumor cells [32, 33, 167, 168, 180, 183]. We have recently demonstrated that both Dp44mT and DpC act to markedly increase NDRG1 expression, which subsequently also led to the inhibition of Src activity, suggesting the therapeutic efficacy of these agents involves the suppression of this oncogene [156]. Notably, DpC is currently under active preclinical development and clinical trials are planned in 2015 [24, 166, 184], which hopefully will lead to the development of new anti-cancer therapeutics in the near future.

As the field grows, and additional novel strategies for therapeutic intervention are developed, the number and kind of targets are likely to increase. Moreover, future candidate metastasis suppressor genes may also prove tractable as pharmacological targets.

## FUTURE PERSPECTIVES AND CONCLUSION

This review has briefly summarized the known molecular interactions between the potent oncogene Src and a variety of metastasis suppressors. However, as discussed, the role of Src in cancer metastasis can be multifaceted, with this latter oncogene being able to negatively regulate a number of metastasis suppressors, while itself being a target for these proteins. This further indicates the complex relationship between Src and metastasis suppressors, which participate in a delicate balance that ultimately determines a cell's ability to invade and metastasize. Importantly, understanding the complex interaction between c-Src and key metastasis suppressors such as NDRG1 has resulted in the development of new anti-metastatic therapies such as the novel agents, Dp44mT and DpC.
